# Supplementary material for: Quasicrystal nucleation and ℤ module twin growth in an intermetallic glass-forming system
Source: Nat Commun. 2018 Oct 3;9:4054. doi: 10.1038/s41467-018-06597-0 (PMC6170452; doi:10.1038/s41467-018-06597-0)
Supplement: Supplementary file 1 — Supplementary Information [file 41467_2018_6597_MOESM1_ESM.pdf]

Quasicrystal nucleation and Z module twin growth in an intermetallic glass-former

W. Hornfeck *et al.*

September 7, 2018

Contents

1 Supplementary Figures **2**

2 Supplementary Tables **12**

3 Supplementary Methods **18**

3.1 Construction of the twin model 18

3.2 Details regarding homogeneous nucleation 19

3.2.1 Skripov analysis 19

3.2.2 Calculation of the critical radius 20

4 Supplementary Discussion **21**

4.1 Remarks on the detailed solidification behavior of NiZr 21

4.1.1 On the competing solidification of CsCl- and CrB-type NiZr 21

4.1.2 Physical interpretation of the temperature-time profile 22

4.1.3 Influence of the solidification behavior on the microstructure 23

4.2 On the glass forming ability of NiZr 23

5 Supplementary References **25**

5.1 References regarding Supplementary Methods and Discussion 25

5.2 Further reading 26

1 Supplementary Figures

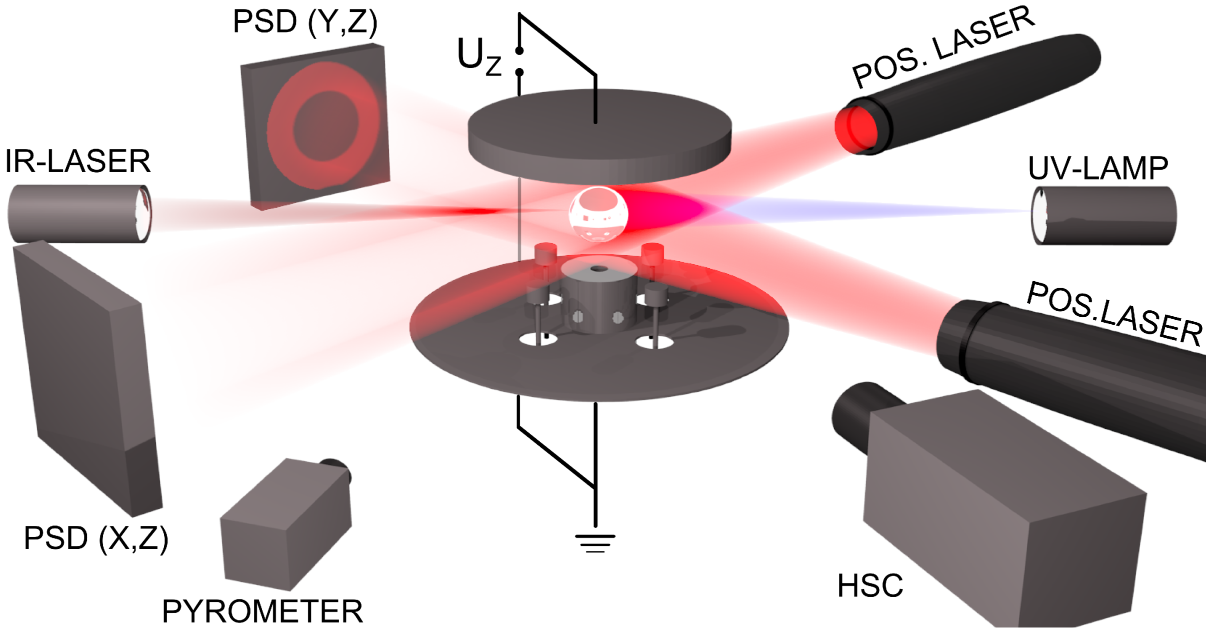

Supplementary Figure 1: **Electrostatic levitation.** Scheme of the experimental setup consisting of a symmetrical designed system of electrodes with a pair (quadruple) of them used for levitation and z-axis (xy-plane) positioning. A sample's position is determined and adjusted in real-time by the shadow of the sample casted during orthogonal illumination with a pair of He/Ne-lasers onto oppositely mounted photosensitive detectors (PSDs). An infrared laser is used for heating the sample, controlled by a contactless pyrometer. Ultraviolet radiation, by inducing a photoelectric effect, is used to keep the surface charge balanced for levitation, prior to melting of the sample. A high-speed camera (HSC) allows direct observation of solidification-related phenomena.

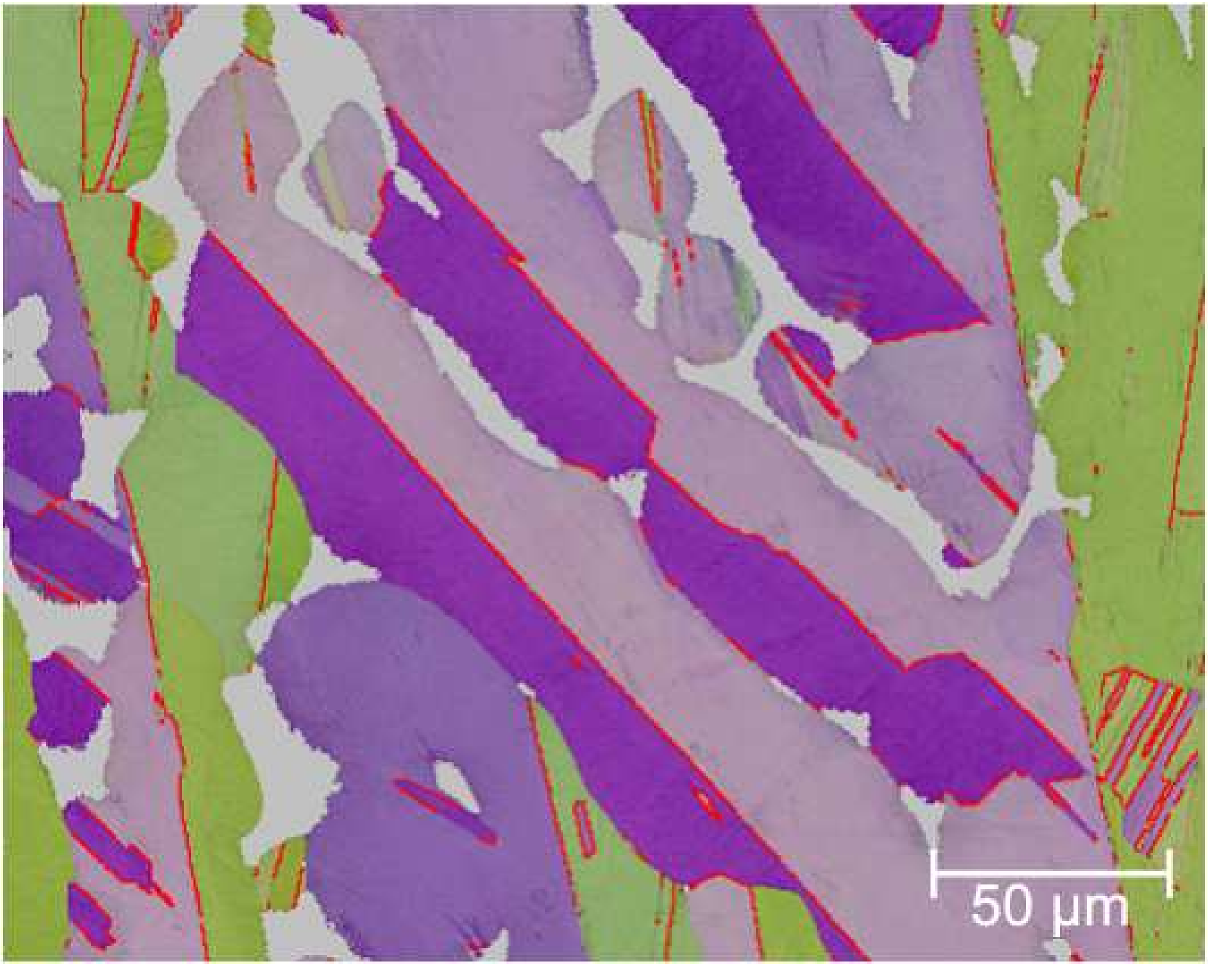

Supplementary Figure 2: Twinned dendrites as primary growth feature. Shown are (coarsened) twinned dendrites, with their coherent (straight) 36° twin boundaries highlighted in red, surrounded by cavities (light gray areas). This proves that twinning is a genuine primary growth feature and not a result of secondary transformations affecting the sample after its solidification. Note the appearance of incoherent (wavy) twin boundaries resulting from the coalescence of neighbouring dendrites yielding an alternating pattern of parallel straight and wavy twin boundaries.

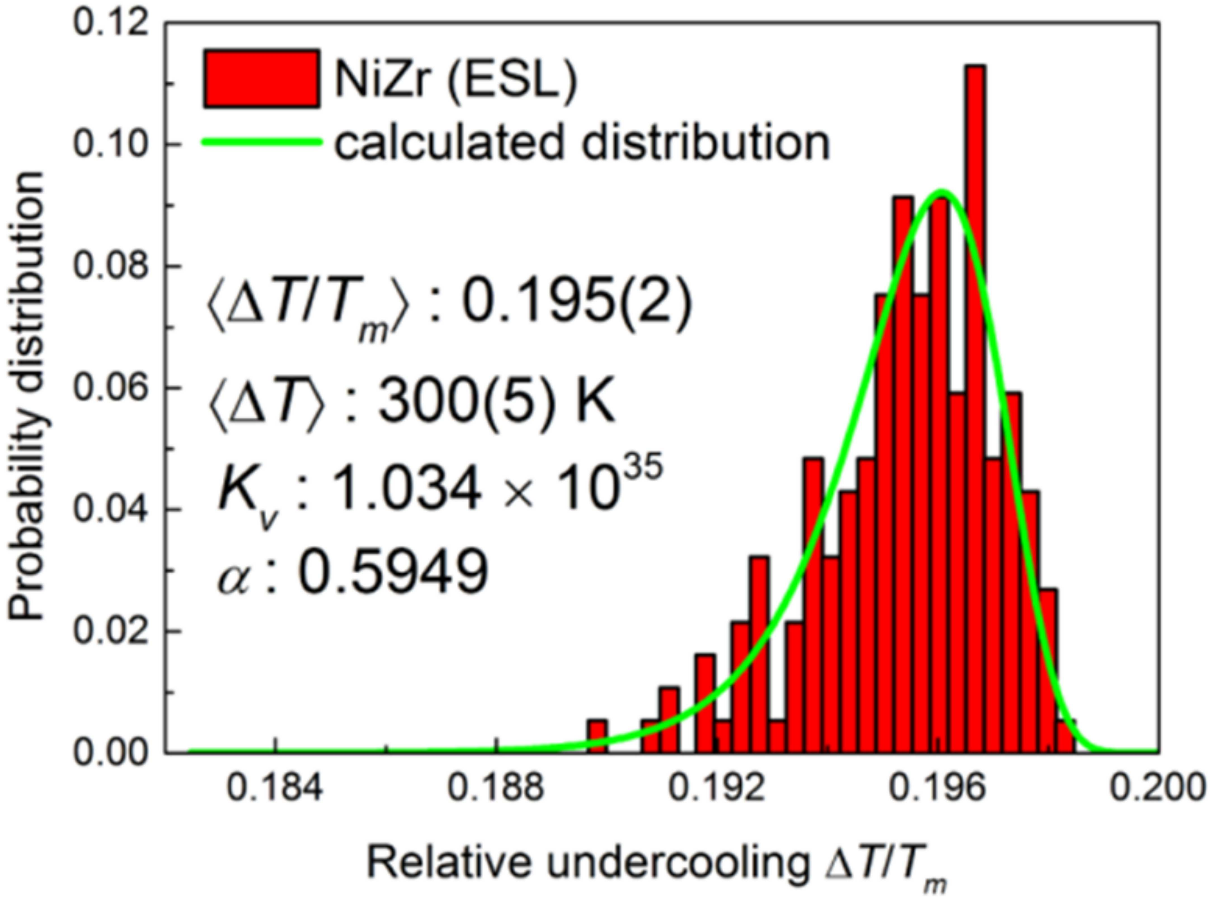

Supplementary Figure 3: Nucleation statistics for ESL-processed NiZr. A smooth distribution function is superimposed onto a histogram showing the normalized frequency of nucleation events versus the relative undercoolings $\Delta T/T_{\mathrm{m}}$ for 200 consecutive solidification cycles with results originating from the statistical analysis.

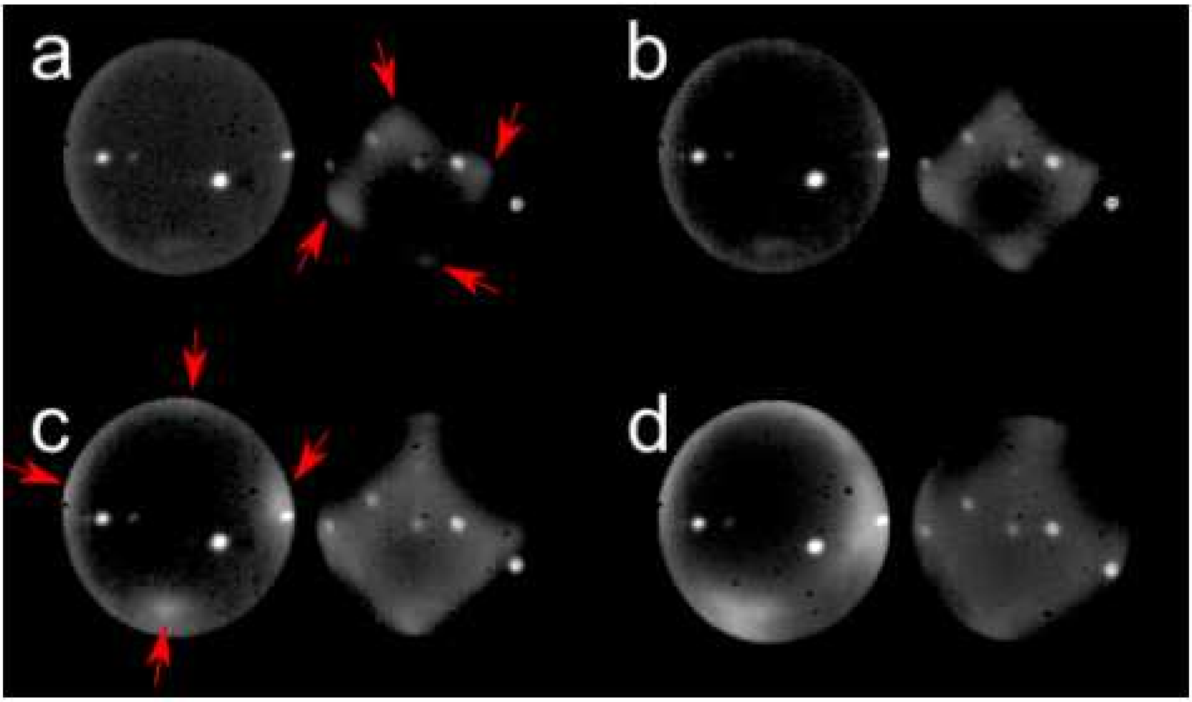

Supplementary Figure 4: Fourfold symmetric solidification front of CsCl-type NiZr solidifying as the primary phase at lower (partly, $\Delta T < 250\,\mathrm{K}$) and lowest (exclusively, $\Delta T < 70\,\mathrm{K}$) undercoolings. The fourfold symmetry exhibited on the surface of the levitated droplet in the early stages of solidification can be traced back to the cubic symmetry of the growing crystal intersecting the surface. Panels **a** to **d** represent different consecutive time steps of the observation of the solidification with arrows highlighting the fourfold symmetry. The picture on the left and right show the front and back side of the levitating droplet, respectively. The diameter of the droplet is about 2.5 mm.

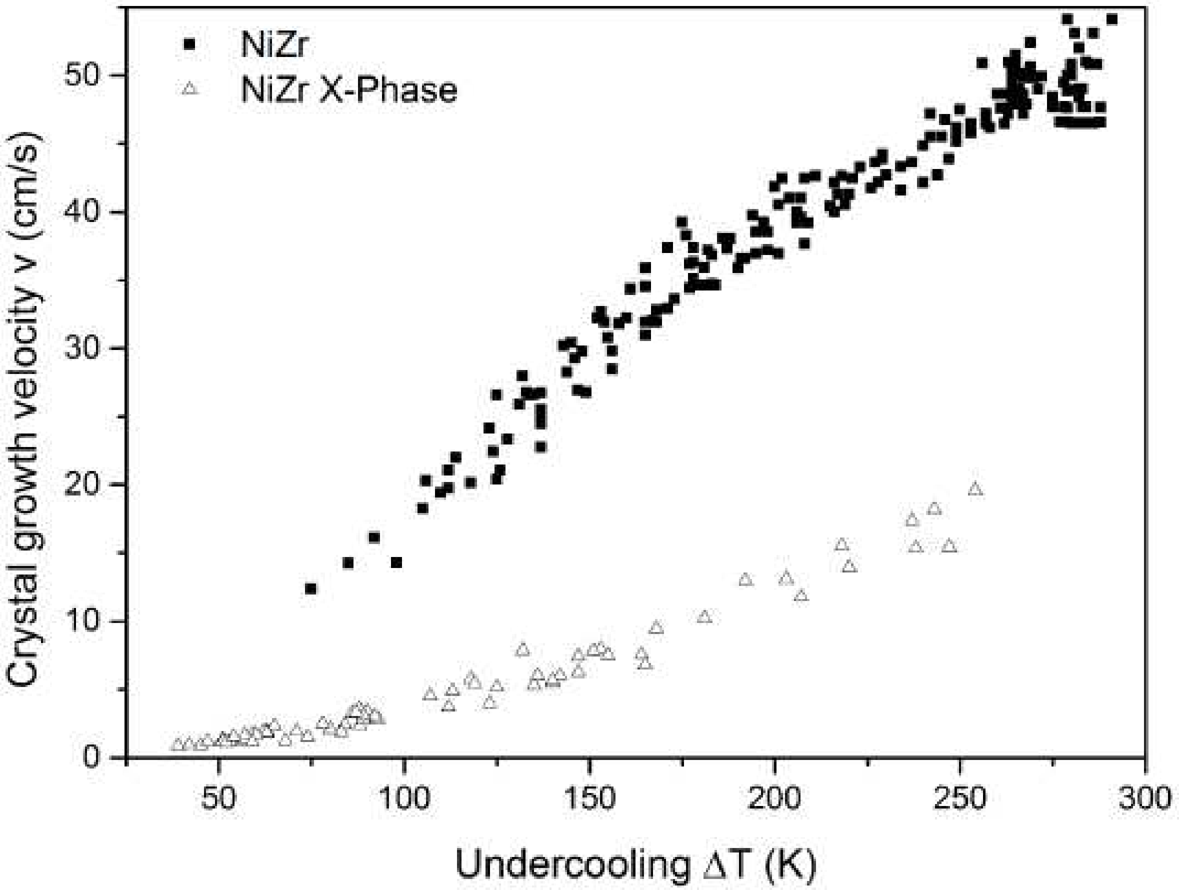

Supplementary Figure 5: Crystal growth velocities of CrB-type NiZr (solid squares) and CsCl-type NiZr (open triangles, here called X-Phase) as a function of undercooling. Note the broad region in which both phases can solidify as the primary phase, and the exclusive regions at lowest and highest undercoolings in which only one phase is observed to solidify in all experiments. CsCl-type (CrB-type) NiZr is the primary phase exclusively occurring at lowest (highest) undercoolings.

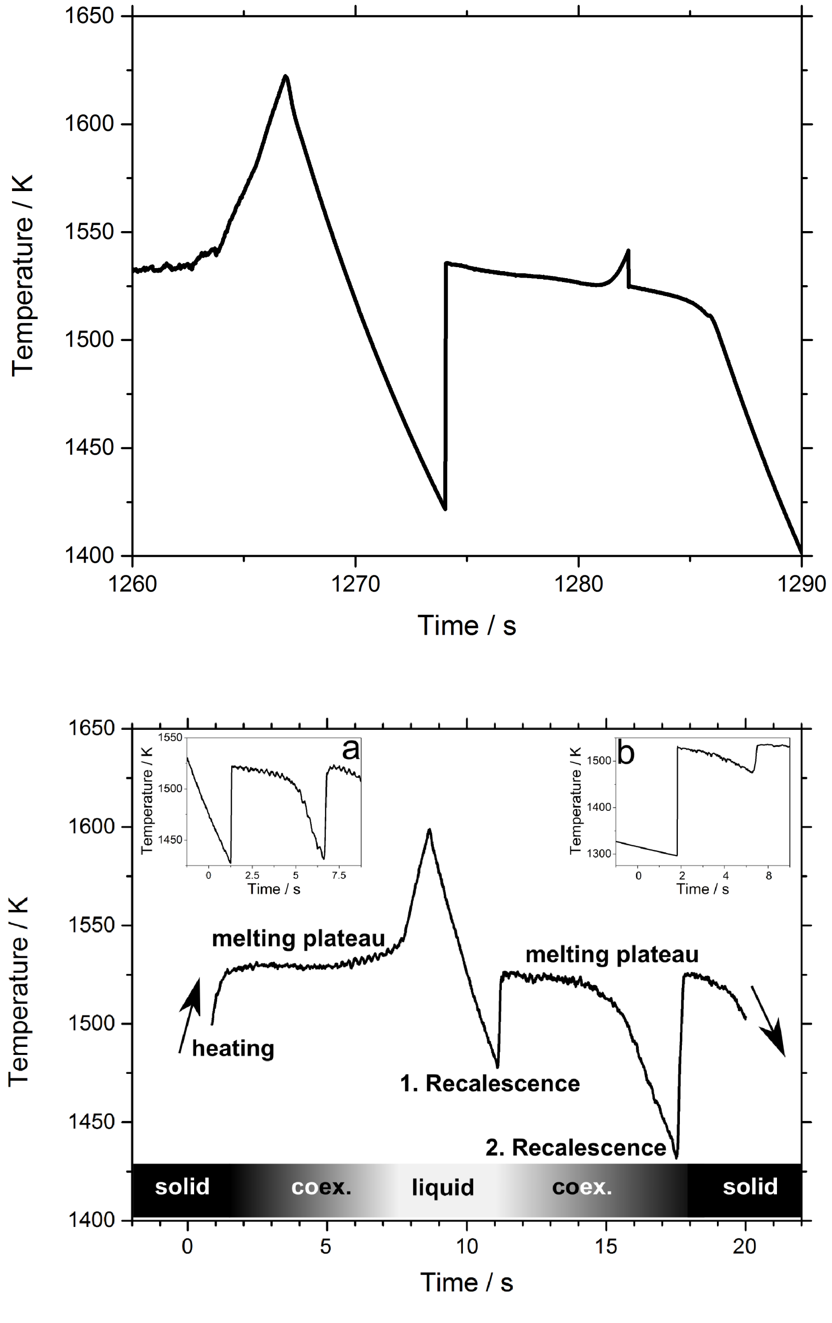

Supplementary Figure 6: Temperature-time profiles for NiZr solidification in the CrB-type (top) and CsCl-type (bottom) structure. Note the occurrence of a double recalescence in the solidification of CsCl-type NiZr. The undercooling values are $\Delta T = 110\,\mathrm{K}$ for CrB-type NiZr (top) and $\Delta T = 108\,\mathrm{K}$ (inset a), $\Delta T = 233\,\mathrm{K}$ (inset b), $\Delta T = 58\,\mathrm{K}$ (main graph) for CsCl-type NiZr (bottom). Note also that for the case of CsCl-type NiZr the magnitude of both recalescences is coupled: if the first one is larger, then the second one will be smaller, and vice versa.

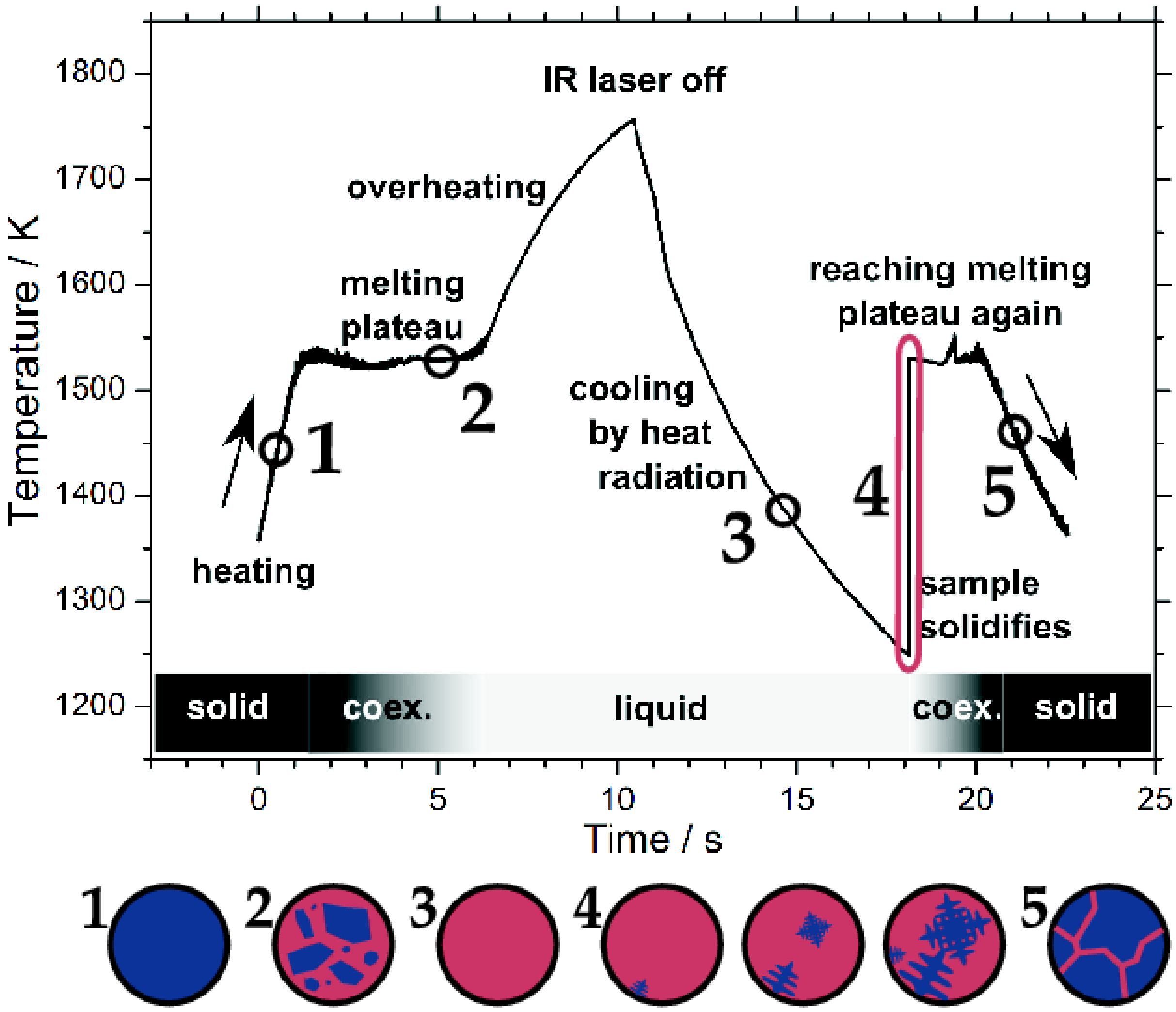

Supplementary Figure 7: Temperature-time profile for NiZr solidification. Processes occuring inside the sample are depicted schematically.

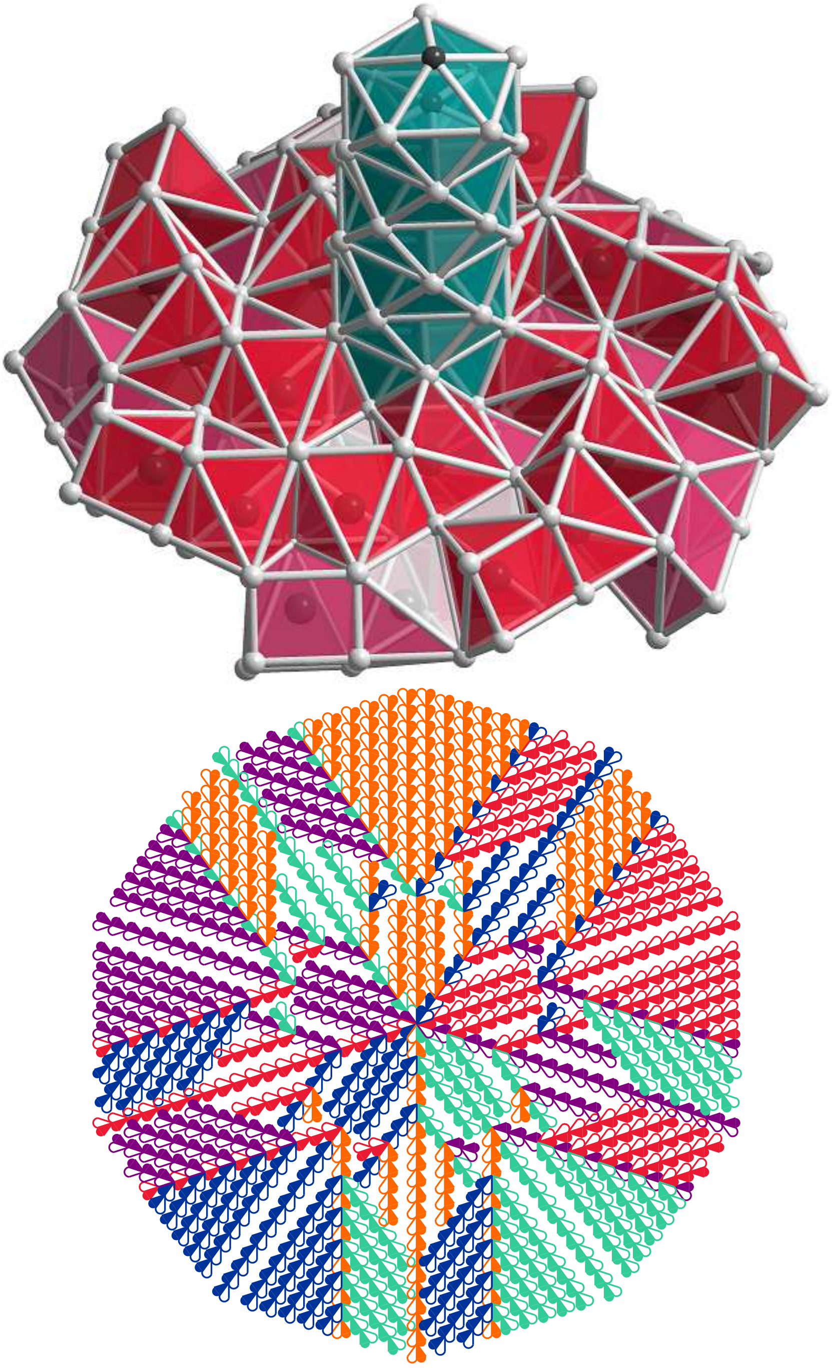

Supplementary Figure 8: Schematic growth features of the tenfold twin The growth of the tenfold twin can be envisaged by the out-of-plane growth of a pentagonal antiprismatic column along [001], amended by a spiral growth in the plane perpendicular to this direction (top). At some moment dendritic solidification is triggered at the kinks of the growing spiral structure, eventually resulting in a tenfold twinned dendritic microstructure (bottom; different orientations highlighted by distinct colors), with its decagonal convex hull mimicking a the solidification front. Parallel straight and wavy twin boundaries and the polar domain structure with wedge-like junctions match the real microstructure even at this level of simplification.

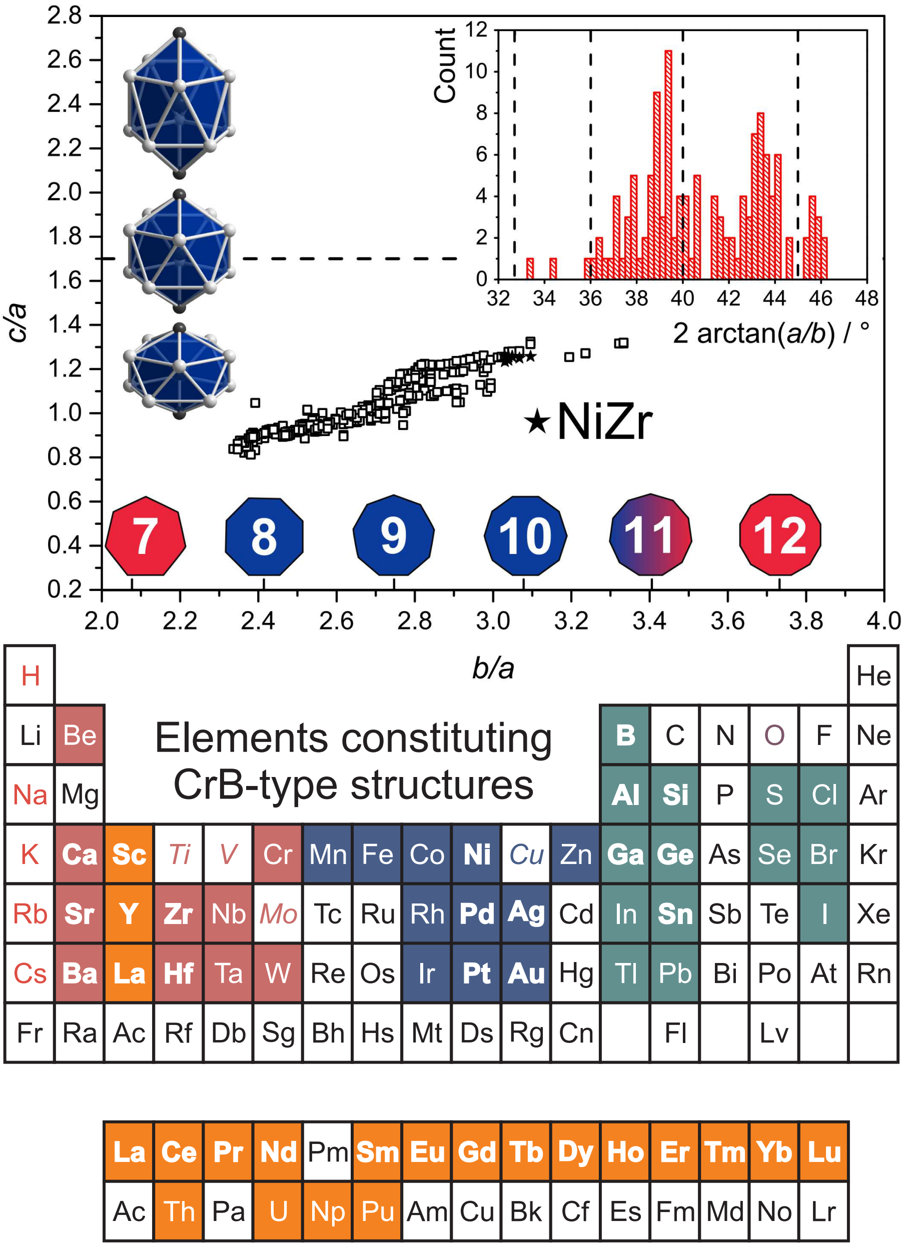

Supplementary Figure 9: Occurrence of 132 binary CrB-type structures regarding crystallo-geometrical constraints and element combinations. The b/a ratio determines the in-plane geometry, i.e. the 'polygonality' of the twin model. The c/a ratio determines its out-of-plane geometry, i.e. the compression/elongation of the central icosahedron, distinguishing between a (2+1)-dimensional, decagonal and a 3D, icosahedral case (top). The majority of CrB-type structures is formed between electropositive alkaline and rare earth elements and early transitions metals in combination with electronegative late transitions metals and main group elements with an additional dependance on the size ratio of the constituents. A special case is given for the alkali metals and the elements hydrogen and oxygen, since the pseudo-binary hydroxides MOH ($M = \mathrm{Na, K, Rb, Cs}$ also crystallize in the CrB-type. The metals Ti, V, and Cu occur only as the minority components of ternary substitution variants of the CrB-type structure. Supplementary Table 4 contains a list of the crystallographic data for those compounds, for which the complete crystal structure, including the atomic coordinates, was determined (bottom).

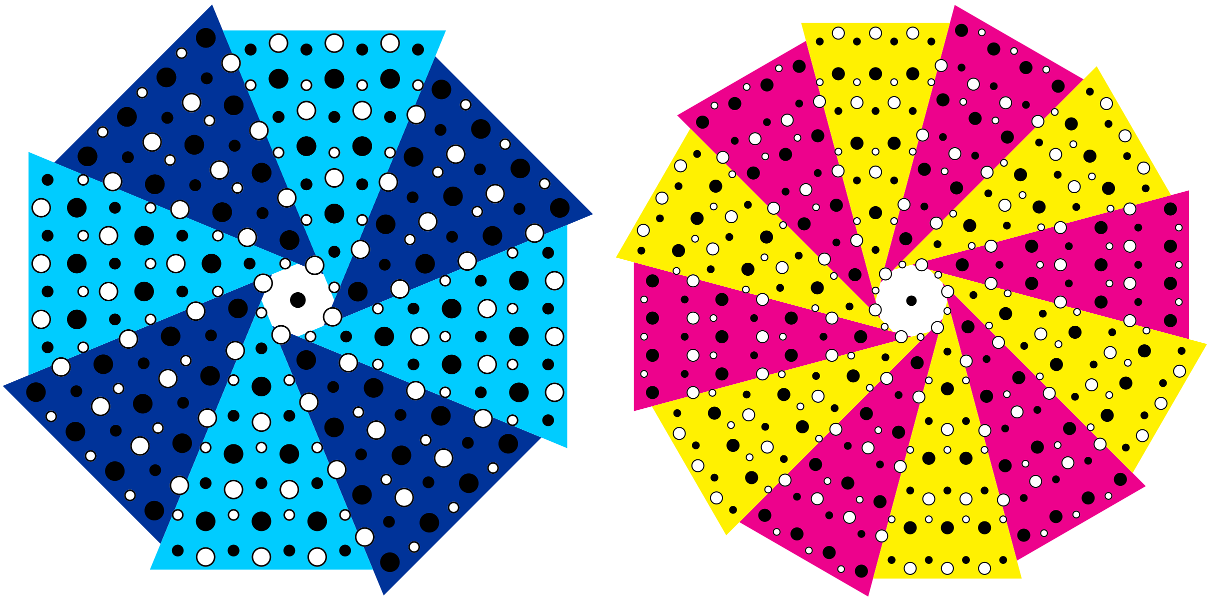

Supplementary Figure 10: Eightfold and twelvefold twinned CrB-type structure variants. Note the opposite chirality of the spirals. See Supplementary Table 3 for the numerical data for these models.

2 Supplementary Tables

Supplementary Table 1: **Glass-forming range of binary Ni–Zr alloys.** Given are the minimal and maximal composition (stated in at.% Zr) in between glass-formation was observed either experimentally or by means of molecular dynamics simulations. In addition to the Supplementary References some details are listed: In the case of experimental data the method used for glass-production and details of the analysis for establishing the state of the sample, as well as simulation details in the case of simulations. Abbreviations: IM = ion beam mixing, MQ = melt quenching (e.g. melt spinning, splat cooling), MA = mechanical alloying, SSAR = solid state amorphization reaction, MD = molecular dynamics, MS = magnetic susceptibility measurements, TEM = transmission electron microscopy, RBS = Rutherford backscattering spectrometry, Auger = Auger depth profiling, X-ray = X-ray diffraction.

min.	max.	NiZr in GFR?	method of synthesis	method of meas./sim.	Suppl. Ref.	remarks
10	80	yes	IM	TEM	[12]	
10	80	yes	MQ	X-ray	[13]	no sample with exact NiZr composition
27	83	yes	MA	X-ray	[14]	
20	67	yes	SSAR	RBS	[15]	
20	80	yes	SSAR	Auger	[16]	
30	80	yes	MA	TEM/X-ray	[17]	
33/60	42/76	no	MQ	X-ray	[18]	two intervals given excluding NiZr
33	52	yes	SSAR	RBS/X-ray	[19]	
35	72	yes	MA	X-ray/MS	[20]	
35	63	yes	SSAR	nanocalorimetry	[21]	$Ni_{55.5}Zr_{44.5}$ predicted good GFA
20	67	yes	–	MS	[22]	local maxima of the GFA at the eutectics
20	85	yes	–	MD	[23]	
14	75	yes	–	MD	[24]	
40	70	no	–	CALPHAD	[25]	only local maxima of the GFA determined
30	80	yes	–	CALPHAD	[26]	amorphous-forming range from ternary phase diagram
17	67	yes	–	CALPHAD	[27]	
33	85	yes	–	MD	[28]	most recent MD study
–	–	yes	–	MD	[29]	most recent MD study focusing on NiZr

Supplementary Table 2: Physical parameters of NiZr used in the evaluation of the nucleation statistics (top) or resulting from it (bottom).

Parameter	Symbol	Value	Unit	Ref.
Liquidus temperature	T_L	1533	K	[1]
Enthalpy of crystallization	ΔH_{cr}	14.73(4)	kJ/mol	[2]
Entropy of crystallization	$\Delta S_{\mathrm{cr}} = \Delta H_{\mathrm{cr}}/T_L$	9.60861	J/(mol K)	calc.
Cooling rate	$\Delta T/\Delta t$	34	K/s	meas.
Sample radius	r	1.29×10^{-3}	m	meas.
Sample volume	$V = (4/3)\pi r^3$	8.95×10^{-9}	m^3	meas.
Molar volume NiZr	$\left(V_{\mathrm{m}} = N_{\mathrm{A}} V_{\mathrm{cr}}/Z\right)$ $\left(V_{\mathrm{m,Ni}} + V_{\mathrm{m,Zr}}\right)/2$	1.03×10^{-5}	m^3/mol	calc.
Critical free enthalpy	ΔG^*	59.717	$k_{\mathrm{B}} T_{\mathrm{n}}$	
Solid-liquid interfacial energy	σ	0.2107(6)	J/m^2	
Dimensionless interfacial energy	α	0.5949	−	
Pre-exponential factor	K_V	1.034×10^{35}	$m^{-3}s^{-1}$	
Critical radius	r^*	1.1	nm	

Supplementary Table 3: Ideal geometric parameters for n-fold twin structures ($n = 8, 10, 12$).

	$n = 8$		
$\varphi/2$		22.5°	
b/a	$1 + \sqrt{2}$	2.414214	
L/a	$1/\sqrt{2}$	0.707107	
y_B/b	$1/(2\sqrt{2})$	0.353553	
S/a	$(-1 + \sqrt{10})/(3\sqrt{2})$	0.509654	
y_A/b	$-(1/12)(-1 + \sqrt{2})(\sqrt{2} - 2\sqrt{5})$	0.105553	
σ/d	$1/(2\sqrt{2(2 + \sqrt{2})})$	0.191342	
	$n = 10$		NiZr
$\varphi/2$		18°	18.2°
b/a	$\sqrt{5 + 2\sqrt{5}}$	3.077684	3.040698
L/a	$\sqrt{(1/10)(5 + \sqrt{5})}$	0.850651	0.868579
y_B/b	$(1/20)(5 + \sqrt{5})$	0.361803	0.3609(8)
S/a	$\sqrt{(1/10)(5 - \sqrt{5})}$	0.525731	0.534687
y_A/b	$(1/20)(-5 + 3\sqrt{5})$	0.085410	0.0817(17)
σ/d	$(1/8)(-1 + \sqrt{5})$	0.154508	
	$n = 12$		
$\varphi/2$		15°	
b/a	$2 + \sqrt{3}$	3.732051	
L/a	1	1.000000	
y_B/b	$(1/2)(-1 + \sqrt{3})$	0.366025	
S/a	$(1/3)(-1 + \sqrt{7})$	0.548584	
y_A/b	$-(1/6)(-2 + \sqrt{3})(-1 + \sqrt{7})$	0.073496	
σ/d	$1/(4\sqrt{2 + \sqrt{3}})$	0.129410	

The following table lists a selection of crystallographic data for those 132 binary CrB-type structures for which the complete crystal structure has been determined.

Supplementary Table 4: Crystallographic data of binary CrB-type structures.

Formula	a/pm	b/pm	c/pm	a/b	c/a	y_A	y_B
AgCa	405.22	1144.70	464.43	0.354	1.146	0.0741(3)	0.35773(10)
AgCl	332.00	983.50	410.80	0.338	1.237	0.102(1)	0.359(1)
AlHf	325.30	1082.20	428.00	0.301	1.316	0.108	0.367
AlTh	442.00	1145.00	419.00	0.386	0.948	0.057	0.353
AlY	388.40	1152.20	438.50	0.337	1.129	0.07	0.35
AlZr	335.30	1086.60	426.60	0.309	1.272	0.076	0.334
AuCa	396.10	1107.50	457.60	0.358	1.155	0.08	0.36
AuCe	390.00	1114.00	475.00	0.350	1.218	0.065	0.365
AuDy	371.00	1087.00	461.00	0.341	1.243	0.065	0.365
AuEr	365.00	1081.00	458.00	0.338	1.255	0.065	0.365
AuGd	376.00	1094.00	464.00	0.344	1.234	0.065	0.365
AuHo	368.50	1083.50	459.50	0.340	1.247	0.065	0.365
AuLa	395.00	1120.00	478.00	0.353	1.210	0.065	0.365
AuNd	384.00	1107.00	470.00	0.347	1.224	0.065	0.365
AuPr	387.00	1110.00	472.00	0.349	1.220	0.065	0.365
AuSm	380.00	1100.00	466.00	0.345	1.226	0.065	0.365
AuTb	373.00	1090.00	462.00	0.342	1.239	0.065	0.365
AuTm	362.00	1078.00	457.00	0.336	1.262	0.065	0.365
BCr	297.82	787.00	293.46	0.378	0.985	0.064(2)	0.35475(3)
BTa	327.60	866.90	315.70	0.378	0.964	0.06	0.354
CoTh	374.00	1088.00	416.00	0.344	1.112	0.084	0.364
CoY	410.60	1035.80	390.60	0.396	0.951	0.068(2)	0.354(1)
GaCa	419.30	1144.70	438.47	0.366	1.046	0.06651(6)	0.35401(11)
GaCe	447.30	1133.90	420.10	0.394	0.939	0.08	0.361
GaDy	430.00	1089.00	406.70	0.395	0.946	0.074	0.36
GaGd	433.88	1101.04	410.58	0.394	0.946	0.0717(1)	0.3583(1)
GaHo	428.10	1077.40	405.00	0.397	0.946	0.0763	0.3579
GaLa	449.30	1134.10	420.80	0.396	0.937	0.083	0.362
GaNd	439.00	1134.00	419.70	0.387	0.956	0.082	0.378
GaPr	446.00	1133.80	419.90	0.393	0.941	0.08	0.359
GaSc	402.20	1020.50	389.50	0.394	0.968	0.083	0.362
GaSm	433.00	1134.00	419.60	0.382	0.969	0.079	0.378
GaTb	433.00	1090.00	409.00	0.397	0.945	0.074	0.359
GaY	430.20	1086.00	407.30	0.396	0.947	0.08300	0.362
GeBa	505.70	1194.20	429.90	0.423	0.850	0.064(3)	0.36(4)
GeDy	425.40	1062.30	390.40	0.400	0.918	0.0773(4)	0.3615(7)
GeEr	421.99	1058.10	390.60	0.399	0.926	0.0859(8)	0.3613(8)

Supplementary Table 4: Crystallographic data of binary CrB-type structures (continued).

Formula	a/pm	b/pm	c/pm	a/b	c/a	y_A	y_B
GeEu	471.50	1126.00	410.10	0.419	0.870	0.071	0.361
GeGd	433.90	1078.80	397.30	0.402	0.916	0.08	0.36
GeNd	447.80	1107.50	404.30	0.404	0.903	0.0798(4)	0.3611(4)
GePr	447.20	1107.00	402.90	0.404	0.901	0.083(1)	0.359(1)
GeS	365.76	956.88	327.23	0.382	0.895	0.11062	0.3581
GeSc	400.70	1006.00	376.20	0.398	0.939	0.083	0.362
GeSm	438.70	1089.00	399.30	0.403	0.910	0.08	0.36
GeSr	480.80	1136.00	416.90	0.423	0.867	0.07(2)	0.362(3)
GeTb	428.26	1068.02	392.90	0.401	0.917	0.0844(5)	0.3611(5)
GeTm	418.50	1052.40	388.50	0.398	0.928	0.06	0.354
GeY	426.20	1069.40	394.10	0.399	0.925	0.083	0.362
HoGe	424.28	1062.30	391.90	0.399	0.924	0.0832(6)	0.3619(6)
InBr	461.31	1271.20	469.96	0.363	1.019	0.111	0.3508
InCl	424.20	1232.00	468.90	0.344	1.105	0.11189(31)	0.34525(8)
InI	451.82	1149.20	418.61	0.393	0.927	0.094	0.3567
NiCe	379.40	1054.60	436.70	0.360	1.151	0.0763	0.36134
NiGd	377.13	1032.72	424.88	0.365	1.127	0.0789(2)	0.3571(5)
NiNd	380.59	1046.20	433.45	0.364	1.139	0.0727(5)	0.36149(1)
NiZr	327.12	993.10	410.72	0.329	1.256	0.084(0)	0.36(0)
PbBa	529.00	1260.00	478.00	0.420	0.904	0.0802(3)	0.3734(8)
PdGd	373.60	1055.00	454.80	0.354	1.217	0.085	0.36
PbSr	501.80	1223.00	464.80	0.410	0.926	0.078	0.368
PdZr	333.05	1030.40	437.45	0.323	1.313	0.0876(2)	0.3572(2)
PrNi	383.07	1054.30	436.90	0.363	1.141	0.0724(1)	0.36178(4)
PtCe	388.40	1084.50	451.70	0.358	1.163	0.0872(7)	0.3638(10)
PtNd	384.60	1076.90	454.20	0.357	1.181	0.084	0.369
PtPr	389.10	1089.90	456.90	0.357	1.174	0.091	0.368
PtTh	390.00	1109.00	445.40	0.352	1.142	0.09(2)	0.36(4)
PtU	370.30	1079.20	439.10	0.343	1.186	0.0889(3)	0.362(2)
PtZr	340.82	1030.00	428.06	0.331	1.256	0.0904(1)	0.3582(1)
PuNi	359.00	1021.00	422.00	0.352	1.175	0.0779(9)	0.3579(34)
RhTh	386.60	1124.00	422.00	0.344	1.092	0.09(2)	0.36(4)
SiBa	495.10	1178.70	401.90	0.420	0.812	0.058	0.358
SiCa	451.92	1067.80	387.34	0.423	0.857	0.0697	0.3616
SiDy	424.72	1047.55	380.08	0.405	0.895	0.0669(7)	0.3586(2)
SiEr	419.50	1035.30	377.90	0.405	0.901	0.087(1)	0.358(2)
SiEu	469.58	1112.42	397.99	0.422	0.848	0.0649(8)	0.36(2)
SiHo	423.13	1044.74	380.54	0.405	0.899	0.077(1)	0.3591(1)
SiSc	398.80	988.20	365.90	0.404	0.918	0.081(1)	0.36(1)
SiSr	482.60	1133.70	405.20	0.426	0.840	0.0639	0.361
SiY	425.10	1052.60	382.60	0.404	0.900	0.06	0.354
SiYb	417.80	1031.00	376.80	0.405	0.902	0.075	0.361
SnBa	531.00	1248.50	465.00	0.425	0.876	0.075	0.368
SnCa	476.61	1153.80	434.52	0.413	0.912	0.0857	0.3669
SnEu	497.60	1190.00	445.60	0.418	0.896	0.076	0.365
SnS	413.60	1148.80	417.20	0.360	1.009	0.1251(1)	0.35(2)
SnSe	430.70	1171.60	430.70	0.368	1.000	0.124(1)	0.356(2)
SnSr	504.50	1204.00	449.40	0.419	0.891	0.0788(4)	0.3633(3)
TlI	457.73	1278.30	516.35	0.358	1.128	0.1079	0.368
ZnCa	420.20	1161.00	444.20	0.362	1.057	0.065	0.355

In the following we give a qualitative overview over ESL-processed samples of binary NiZr, NiHf and CuZr as well as ternary (Ni,Cu)Zr and Ni(Zr,Hf) focusing on the presence (+) or absence (−) of features indicating n-fold twinning in either HSC-videos, optical micrographs (observing geodesics; OM), EBSD-maps or IPF-plots (n.d. = not determined). Less distinctive, yet still detectable features are denoted by a bracketed symbol.

Supplementary Table 5: Selection of ESL-processed samples.

Sample	$\Delta T/K$	n	HSC	OM	EBSD	IPF	Remarks
NiZr	93, 108	10	−	−	−	−	irregular front
NiZr	180		−	+	−	+	polygonal front, poles visible
NiZr	203, 285		+	+	+	+	polygonal front, poles visible
NiZr	300		+	+	+	+	
$Ni_{53.5}Zr_{46.5}$	229	10	−	−	−	+	possible 4-fold front (cubic B2 phase)
$Ni_{49.1}Zr_{50.9}$	271		+	(+)	n.d.	n.d.	10 vague longitudes
$Ni_{47.6}Zr_{52.4}$	272		+	(+)	(+)	(+)	10-fold in small regions
$Ni_{45.3}Zr_{54.7}$	257		−	−	(+)	(+)	10-fold in small regions
$Ni_{50}(Zr_{49}Hf)$	291	10	+	+	+	+	−
$Ni_{50}(Zr_{47.5}Hf_{2.5})$	294		+	+	n.d.	n.d.	2nd phase visible (HSC)
$Ni_{50}(Zr_{45}Hf_5)$	67	10	−	−	−	−	4-fold front (cubic B2 phase)
$Ni_{50}(Zr_{45}Hf_5)$	95		−	−	n.d.	n.d.	4-fold front (cubic B2 phase)
$Ni_{50}(Zr_{45}Hf_5)$	288		+	+	n.d.	n.d.	2nd phase visible (HSC)
$Ni_{50}(Zr_{40}Hf_{10})$	310	10	−	(+)	(−)	(+)	10 vague longitudes
NiHf	330, 429	10	−	−	−	+	4-fold front (cubic B2 phase)
$(Ni_{45}Cu_5)Zr_{50}$	81	10	−	−	n.d.	n.d.	4-fold front (cubic B2 phase)
$(Ni_{45}Cu_5)Zr_{50}$	306		+	(+)	n.d.	n.d.	vague geodesics
$(Ni_{30}Cu_{20})Zr_{50}$	99, 187	10	−	−	n.d.	n.d.	4-fold front (cubic B2 phase)
$(Ni_{30}Cu_{20})Zr_{50}$	402		−	−	n.d.	n.d.	spherical front
$(Ni_{20}Cu_{30})Zr_{50}$	99, 174	10	−	−	n.d.	n.d.	possible 4-fold (cubic B2 phase)
$(Ni_{20}Cu_{30})Zr_{50}$	377	10	−	−	n.d.	n.d.	spherical front
$(Ni_5Cu_{45})Zr_{50}$	65, 346	10	−	−	n.d.	n.d.	spherical front
CuZr	104, 297	10	−	−	n.d.	n.d.	spherical front
PdZr	400	10?	−	−	−	−	B2-phase; martensitic transition
PtZr	336	10?	−	−	−	−	4-fold front; $\Delta T = 514\,K$ to reach CrB type phase
NiB	108	8	−	+	+	+	domains/center visible; no high undercoolings were reached
NiGd	n.d.	9?	−	−	−	−	no undercooling in ESL
SiZr	n.d.	9?	−	−	−	−	melting unsuccessful in ESL
CoZr	416	12?	−	+	+	+	CrB type found; domains/center vis.

3 Supplementary Methods

3.1 Construction of the twin model

For a compound AB crystallizing in the orthorhombic CrB-type structure with lattice parameters a, b, c we define a pair of distances

$$S = \sqrt{d_{\mathrm{AA}}^2 - (c/2)^2} \qquad \text{and} \qquad L = \sqrt{d_{\mathrm{BB}}^2 - (c/2)^2}\,, \tag{1}$$

where d_{AA} and d_{BB} are the shortest interatomic distances between alike constituents (here, A = Ni and B = Zr). In fact, S and L represent *projections* – along the c-direction – of the distances d_{AA} and d_{BB}, respectively. Another pair of distances is given by

$$S' = \sqrt{\left(\frac{a}{2}\right)^2 + \left(\frac{L}{2} - \frac{S}{2}\right)^2} \qquad \text{and} \qquad L' = \sqrt{\left(\frac{a}{2}\right)^2 + \left(\frac{b}{2} - L\right)^2}\,. \tag{2}$$

For the construction of the ideal n-fold twinned structure model we impose the restrictions

$$S' \stackrel{!}{=} S \Rightarrow \text{equation (8)} \qquad \text{and} \qquad L' \stackrel{!}{=} L \Rightarrow \text{equation (6)}\,, \tag{3}$$

which allow to derive a complete and consistent set of structural parameters via elementary geometrical relations:

$$\varphi/2 = \frac{360^\circ}{2\,n}\,, \tag{4}$$

$$b = \frac{1}{\tan\varphi/2}\,a\,, \tag{5}$$

$$L = \frac{a^2 + b^2}{4\,b}\,, \tag{6}$$

$$y_{\mathrm{B}} = \frac{b - L}{2\,b}\,, \tag{7}$$

$$S = \frac{1}{3}\left(-L + \sqrt{3\,a^2 + 4\,L^2}\right)\,, \tag{8}$$

$$y_{\mathrm{A}} = \frac{S}{2\,b}\,, \text{ and} \tag{9}$$

$$\sigma = \frac{a}{2} = \frac{d}{2\,\sqrt{1 + (b/a)^2}}\,. \tag{10}$$

This includes special values for the atomic coordinates y_{A} and y_{B} of the Wyckoff position $4c$ $(0, y, 1/4)$ for both constituents. Note, that all parameters given in succession solely depend on the number n of twin domains and refer to a common arbitrary scaling factor given by the lattice parameter a (which may be set to unity). Note also, that for the special case of $n = 10$ (NiZr) the geometrical match is perfect, such that the crystal structure across the twin boundaries is identical to the crystal structure of bulk NiZr. Supplementary Table 3 lists the calculated values for the parameters φ, b, L, y_{B}, S, y_{A}, and σ as defined above for different choices of $n = 8, 10, 12$, with $a = 1$, in analytical and numerical form.

3.2 Details regarding homogeneous nucleation

3.2.1 Skripov analysis

In order to distinguish between heterogeneous or homogeneous nucleation taking place in an undercooled melt, a statistical analysis of nucleation events can be performed, if a single sample is repeatedly molten, undercooled and solidified with its relative undercoolings $\Delta T/T_\mathrm{m}$ determined. A histogram mapping the (normalized) frequency of nucleation events versus $\Delta T/T_\mathrm{m}$ allows the extraction of key parameters of nucleation and eventually the distinction between the heterogeneous and the homogeneous case. For this purpose it is necessary to describe the distribution function of nucleation events by some physical model. Following Skripov's [3] assumption of Poisson-distributed nucleation events a probability distribution

$$\omega(T) = \frac{I_\mathrm{ss} V}{\dot{T}} \exp\left(- \int_{T_\mathrm{L}}^{T} \frac{I_\mathrm{ss} V}{\dot{T}} dT \right) \tag{11}$$

is derived ($\dot{T}$ denoting the time derivative of the temperature). Here, I_ss is the unknown nucleation rate, which can be modelled, using the approximation [4] $\Delta G_V = \Delta S_\mathrm{f}\, \Delta T\, V_\mathrm{m}^{-1}$, as

$$I_\mathrm{ss} = K_V \cdot \exp\left(- \frac{\Delta G^*}{k_\mathrm{B} T_\mathrm{n}} \right) , \tag{12}$$

with T_n the nucleation temperature and the pre-exponential factor

$$K_V = \frac{k_\mathrm{B} T_\mathrm{n}}{3\, a_0^3\, \eta(T)} N_0 \tag{13}$$

of the nucleation rate. Here, a_0 denotes a typical (nearest-neighbour) interatomic distance, $\eta(T)$ is the temperature-dependent dynamical viscosity and N_0 gives the number of nuclei, with $N_0 = N_\mathrm{A}/V_\mathrm{m}$ in the limit, in which all N_A atoms within the molar volume V_m act as nuclei. K_V thus represents a sensitive measure for the degree of homogeneous nucleation.

Now, the cumulative distribution function

$$F(T) = 1 - \exp\left(- \frac{V}{\dot{T}} \int_{T_\mathrm{L}}^{T} I_\mathrm{ss} dT \right) \tag{14}$$

resulting from the integration of $\omega(T)$, is simplified by neglecting the temperature dependence of K_V and written as

$$F(T) = 1 - \exp\left(- \frac{V K_\mathrm{V}}{\dot{T} \frac{d\left(\Delta G^*/(k_\mathrm{B}\, T)\right)}{dT}} \int_{T_\mathrm{L}}^{T} \left(\frac{C T^2}{\Delta T^2} \right) \right) \tag{15}$$

with $-C\, T^2/\Delta T^2 = -\Delta G^*/k_\mathrm{B} T_\mathrm{n}$.

Plotting $\ln(-\ln(1 - F(T)))$ versus $T^2/\Delta T^2$ leads to a linear relation from which K_V and ΔG^* are determined (compare Supplementary Table 2).

3.2.2 Calculation of the critical radius

According to classical nucleation theory (CNT) the critical radius r^* is given as

$$r^* = \frac{2\,\sigma}{\Delta G_{\mathrm{sl}}} \tag{16}$$

where

$$\Delta G_{\mathrm{sl}} = G_{\mathrm{s}} - G_{\mathrm{l}} = \sqrt{\frac{16\,\pi\,\sigma^3}{3\,\Delta G^*}} \tag{17}$$

denotes the difference in the free enthalpy between the solid and the liquid phase. Thus, r^* can be calculated, if the solid-liquid interfacial energy σ and the critical free enthalpy of nucleation ΔG^* are known. While ΔG^* is obtained in the aforementioned way, the solid-liquid interfacial energy σ is calculated based on the negentropic model of Spaepen [5] according to

$$\sigma = \alpha \frac{\Delta H_{\mathrm{cr}}}{\left(N_{\mathrm{A}} V_{\mathrm{m}}^2\right)^{1/3}}\,. \tag{18}$$

Here, ΔH_{cr} denotes the enthalpy of crystallization and α is the dimensionless interfacial energy. Supplementary Table 2 lists the parameters used for and obtained from the statistical evaluation of a series of nucleation events.

4 Supplementary Discussion

4.1 Remarks on the detailed solidification behavior of NiZr

4.1.1 On the competing solidification of CsCl- and CrB-type NiZr

In our manuscript we have focused on the solidification behavior of NiZr at the highest achievable undercoolings. It should be noted, that the solidification behavior at different, lower undercoolings is not the same! Moreover, the solidification behavior also depends on the processing conditions of a sample, sometimes including its thermal history. In particular, this applies to the question if CrB-type (Strukturbericht B33) NiZr is always the primary phase to solidify, which cannot be answered in the affirmative, since a metastable polymorph of CsCl-type (B2) NiZr was observed in an *in situ* X-ray diffraction experiment.[6] However, from this observation no conclusion can be drawn that the solidification pathway *always* includes this metastable phase as the primary phase to nucleate and grow.

In our own experiments we have observed the CsCl-type modification of NiZr as the primary and solely solidifying phase at all undercooling values below 70 K. Its cubic symmetry shows its evident traces in a fourfold symmetric solidification front, as seen in the high-speed camera recording accompanying each solidification run (Supplementary Figure 4). Above undercooling values of 70 K until about 250 K CsCl-type NiZr solidifies in the minority of cases, while CrB-type NiZr is observed in about eight out of ten runs. For undercooling values greater than 250 K CrB-type NiZr is the primary and solely solidifying phase. With an undercooling value of $\Delta T \approx 200\,\mathrm{K}$ the ESL experiment by Quirinale *et al.*[6] lies exactly in the range where both CsCl- and CrB-type NiZr can be observed as the primary solidifying phase, in particular, if a series of repeated solidification runs is performed.

In order to understand the complicated solidification behavior of NiZr our experiments were performed several hundred times, both serially at the highest undercooling value achievable, in order to perform the nucleation statistics as presented in the manuscript, but also individually across the entire range of undercooling values, in order to obtain reliable velocity data for the movement of the solidification front as a function of the undercooling (Supplementary Figure 5).[7, 8]

The case for CrB-type NiZr as the primary solidifying phase at the highest achievable undercooling of $\Delta T = 300(5)\,\mathrm{K}$ is made by the direct observation of the tenfold symmetric solidification front morphology resulting in a tenfold pattern of geodesics at the surface of the sample. Accordingly, CsCl-type NiZr as the primary phase shows a fourfold symmetric solidification front later showing the full cubic symmetry (Supplementary Figure 4). Also in terms of crystal growth velocity (Supplementary Figure 5) and with respect to the occurence of a single or double recalescence (Supplementary Figure 6) both the CrB- and CsCl-type phases can be unanimously differentiated in their occurring as the primary phase.

4.1.2 Physical interpretation of the temperature-time profile

Even at the maximum undercooling achieved the release of latent heat during recalescence does not surpass the hypercooling limit, as defined by the compound's enthalpy of formation. From an graphical extrapolation of the undercooling curve to the onset point of the sample's final cooling curve it can be estimated that only 80 % of the sample solidifies in the recalescence step. Thus, there is 20 % of remaining melt, which solidifies not in the first extremely fast (about 4 ms) recalescence, but in the later course of the solidification process. Principally one can imagine several distinct processes to happen: the non-equilibrium solidification of the melt either into the CsCl- (i) or the CrB-type (ii) primary phase; the equilibrium solidification of the CsCl-type phase from the melt (iii) as a high-temperature phase to the CrB-type, as it is known for the chemically very similar, dimorphic NiHf system; the equilibrium phase transformation of the high-temperature CsCl-type phase into the low-temperature CrB-type phase (iv). We think, based on our combined observations, that the recalescence has to be attributed to the case (ii), with (iii) possibly happening coinciding with the small kink observed in the temperature-time diagram, while (iv) follows during the cooling of the sample to ambient temperature. Experimental observations that suggest such a picture are the direct high-speed camera observation of a second front of latent heat traversing the sample until the kink can be observed in the temperature-time diagram, as well as the change of signal noise coinciding with the kink, from a smooth curve without noise, caused by the still partly molten surface, to a non-smooth curve with noise, representing the completely solidified sample with its rough surface structures (geodesics) rotating in front of the pyrometer thereby causing small emissivity changes, which are represented as noise in the temperature-time diagram. Furthermore, these observations are in accordance with studies on the solidification of binary Ni–Hf and ternary Ni–(Zr,Hf) alloys. The chemically similar Ni–Hf binary alloy system contains a CsCl-type NiHf high-temperature phase, which shows a very similar solidification behavior like the one observed for NiZr. Here it can be observed, that the onset of the kink is moving along the temperature-time diagram as a function of the sample's composition, which establishes the kink as related to a physical effect occurring within the sample, and not, for instance, an artifact of the temperature measurement, e.g. connected with a change in the sample's emissivity. However, it should be noted that the existence of the kink in our Tt-diagrams has no conclusive physical interpretation. We are here in the same situation as Quirinale *et al.*[6] which, as a conclusion to their *in situ* (!) X-ray scattering experiment of the process, write: *There is a spike in the thermal data which appears to coincide with the initial appearance of B33, and is consistently observed in all runs; however, it is uncertain, whether this spike represents the signature of an actual physical process, or is simply an artifact of the measurement technique and subsequent analysis.* It should be noted that some molecular dynamics studies predict CrB-type NiZr as the phase primarily nucleating from the melt, too.[9]

An extensive study about the solidification behavior of NiZr, which is much more complicated in detail than presented here, summarizing and extending the aforementioned topics in much more detail, can be found in the PhD thesis of Raphael Kobold.[8]

4.1.3 Influence of the solidification behavior on the microstructure

Finally, we would like to discuss potential alternative growth mechanism based on (i) the primary nucleation of CsCl-type NiZr and its subsequent solid–solid transformation into CrB-type NiZr, or (ii) the secondary nucleation and growth of CrB-type NiZr on already existent CsCl-type NiZr. Another mechanism (iii) was proposed by Quirinale *et al.*,[6] which is based on a solid–solid transformation triggered by uniaxial tensile stress acting through included liquid droplets in the almost completely solidified CsCl-type NiZr regulus, which are thought to exert a negative pressure on their surroundings, since the density of the CsCl-type phase is higher than that of the melt.

While a displacive transition of the CsCl- into the CrB-type structure seems plausible from geometric considerations, and has to be assumed to exist in the case of dimorphic NiHf, with a CsCl-type high-temperature phase and a CrB-type low-temperature phase existing in the phase diagram, it should be noted that in our case of highly undercooled NiZr *none* of the assumed mechanisms, involving the CsCl-type phase in one way or the other, is able to explain the observed microstructure! In fact, they all fail to do so on the ground of the observed distribution of crystalline domains in the twinned sample. For our case this could be denoted as 10+1, ten $\langle 110 \rangle_{\mathrm{CrB}}$ directions in a plane, explained by the special metrical relations of the CrB-type unit cell, with one common [001] axis normal to this plane. Exactly this (2+1)-dimensional, (10+1)-orientational symmetry is inherently inscribed in the compressed icosahedron of our model. If cubic CsCl-type NiZr would be the seed, why does one *not* observe crystal orientations according to multiplicities of 6, 8, 12, 24, or 48, the latter multiplicity being the highest order of the cubic point group? Or, for that matter, 10+1 oriented domains, but now in as many as different orientations according to these cubic multiplicities? This should be the case even for mechanism (iii), since while the tensile stress would affect only one axis of the crystal structure at a time, its eigensymmetry is isotropic in the first place and would affect all of the twelve symmetry equivalent $\langle 110 \rangle_{\mathrm{CsCl}}$ directions of the CsCl-type phase without any preference.

4.2 On the glass forming ability of NiZr

Apart from the (micro-)structural investigations presented in the main body of our manuscript, a motivation for the choice of NiZr to be the subject of a detailed study of its solidification, was its glass-forming ability (GFA) reported in the literature, and in particular the question if its GFA influences the dendrite growth velocity as a function of undercooling. While the dendrite growth velocity should rise in general with higher undercooling values achieved for the sample, it might be expected that a GFA-dependent influence could exist for the highest undercooling values, effectively lowering the diffusion constant at temperatures closer to the glass transition. This has to be seen in a general context of glass-forming alloys and with respect to a similar behavior expected for the CuZr.

Interestingly, CuZr and NiZr, while chemically similar, do show pronounced differences in their general solidification behavior, as well as in their GFA. In the case of the Ni–Zr alloys,

it is even difficult to measure the glass transition temperature, which is the reason why it is usually not detected on a differential scanning calorimetry (DSC) heating curve. However, it can be measured by other means, namely by measuring the thermomechanical response of a sample under continuous heating and as a function of heating rate and applied stress. [10] ([11] states glass temperatures of 663 K and 666 K for Ni_xZr_{1-x} alloys with $x = 0.47$ and $x = 0.52$, respectively, which are extrapolated from an endothermic heat effect preceding the exothermic crystallization effect(s) in the DSC measurement curves). Moreover, apart from this direct experimental proof for equiatomic NiZr, a multitude of studies was concerned with the GFA of Ni–Zr alloys in general, and tried to establish the glass-forming range (GFR) of these alloys.

We have tried to summarize these studies in Supplementary Table 1 (which is possibly still not an exhaustive listing), taking into account the different approaches to the problem, whether regarding the experimental procedures or with respect to the theoretical methods employed. Of course, such a table can only give a glimpse into the topic and interested readers are referred to the primary literature, as cited below. However, based on the synoptic view on this multitude of results, we take the following conclusions:

1. The GFA of binary Ni–Zr alloys is in general much lower than that of binary Cu–Zr alloys, which are good glass formers, including CuZr;

2. Binary alloys in the Ni–Zr system show a GFA in a broad composition range, including the compound NiZr (except for one non-recent study [17]);

3. The GFA ability is comparatively higher for the eutectic compositions neighboring NiZr in the binary phase diagram, than for NiZr itself;

4. It is possible to measure a glass transition temperature for NiZr, and therefore NiZr is a glass-former [10, 11].

It should be obvious that the phenomenon of glass formation strongly depends not only on the thermodynamics of the alloy system under investigation, but is critically influenced by kinetic effects, explaining the differences in the stated GFR and the dependency on the results on certain methods of choice. For a comparison of the Ni–Zr and Cu–Zr GFA, see [30], and for insights into the GFA of CuZr, see [31].

5 Supplementary References

5.1 References regarding Supplementary Methods and Discussion

Skripov analysis

[1] Nash, P. & Jayanth, C. S. The Ni-Zr (Nickel-Zirconium) system. *Bull. Alloy Phase Diagr.* **5**, 144– 148 (1984).

[2] R. Kobold. Crystal growth in undercooled melts of glass forming Zr-based alloys. PhD thesis. Ruhr University Bochum, 2016. Online open access (in english) at: `https://hss-opus.ub.ruhr-uni-bochum.de/opus4/frontdoor/index/index/docId/4938`.

[3] Skripov V. P., Baidakov, V. G., Kaverin, A. M. Nucleation in superheated argon, krypton and xenon liquids. *Physica A* **95**, 169–180 (1979).

[4] Turnbull, D. Formation of crystal nuclei in liquid metals. *J. Appl. Phys.* **21**, 1022–1028 (1950).

[5] Spaepen, F. A structural model for the solid-liquid interface in monatomic systems. *Acta Met.* **23**, 729–743 (1975).

Remarks on the detailed solidification behavior of NiZr

[6] D.G. Quirinale, G.E. Rustan, S.R. Wilson, M.J. Kramer, A.I. Goldman, M.I. Mendelev. Appearance of metastable B2 phase during solidification of $Ni_{50}Zr_{50}$ alloy: electrostatic levitation and molecular dynamics simulation studies. *J. Phys.: Condens. Matter* **27**, 085004 (6pp) (2015).

[7] R. Kobold, W.W. Kuang, H. Wang, W. Hornfeck, M. Kolbe, D.M. Herlach. Dendrite growth velocity in the undercooled melt of glass forming $Ni_{50}Zr_{50}$ compound. *Phil. Mag. Lett.* **97**, 249–256 (2017).

[8] R. Kobold. Crystal growth in undercooled melts of glass forming Zr-based alloys. PhD thesis. Ruhr University Bochum, 2016. Online open access (in english) at: `https://hss-opus.ub.ruhr-uni-bochum.de/opus4/frontdoor/index/index/docId/4938`.

[9] S. R. Wilson, M. I. Mendelev. Anisotropy of the solid–liquid interface properties of the Ni–Zr B33 phase from molecular dynamics simulation. *Phil. Mag.* **95**, 224–241 (2015).

On the glass forming ability of NiZr

[10] W.-N. Myung, H.-G. Kim, T. Masumoto. Glass transition behaviour of Zr- and Ti-based binary amorphous alloys. *Mater. Sci. Eng. A* **179/180** (1994) 252–255.

[11] K. H. J. Buschow. Short-range order and thermal stability in amorphous alloys. *J. Phys. F: Met. Phys.* **14** (1984) 593–607.

[12] J. Bøttiger, K. Dyrbye, K. Pampus, R. Poulsen. Phase formation during ion beam mixing of transition-metal multilayers. *Phil. Mag. A* **59** (1989) 569–580.

[13] K. H. J. Buschow, N. M. Beekmans. Thermal stability and electronic properties of amorphous Zr-Co and Zr-Ni alloys. *Phys. Rev. B* **19** (1979) 3843–3849.

[14] J. Eckert, L. Schultz, E. Hellstern, K. Urban. Glass-forming range in mechanically alloyed Ni-Zr and the influence of the milling intensity. *J. Appl. Phys.* **64** (1988) 3224–3228.

[15] J. C. Barbour. Diffusivity of Ni in an Amorphous Ni-Zr alloy. *Phys. Rev. Lett.* **55** (1985) 2872–2875.

[16] B. M. Clemens. Amorphous Zirconium-Nickel films formed by solid state reactions. *J. Non-Cryst. Solids* **61/62** (1984) 817–822.

[17] F. Petzold, B. Scholz, H.-D. Kunze. Formation of Amorphous Ni–Zr Powders by Mechanical Alloying. *Mat. Sci. Eng.* **97** (1988) 25–29.

[18] Y. D. Dong, G. Gregan, M. G. Scott. Formation and stability of Nickel–Zirconium glasses. *J. Non-Cryst. Solids* **43** (1981) 403–415.

[19] A. Thomä. Solid state amorphisation reaction in sputtered and evaporated Ni/Zr multilayer films. *J. Phys.: Condens. Matter* **2** (1990) 3167–3175.

[20] A.W. Weeber, H. Bakker. Extension of the glass-forming range of Ni-Zr by mechanical alloying. *J. Phys. F: Met. Phys.* **18** (1988) 1359–1369.

[21] E. Perim, D. Lee, Y. Liu, C. Toher, P. Gong, Y. Li, W.N. Simmons, O. Levy, J.J. Vlassak, J. Schroers, S. Curtarolo. Spectral descriptors for bulk metallic glasses based on the thermodynamics of competing crystalline phases. *Nature Comm.* **7** (2016) 12315 (9pp).

[22] E. Babić, R. Ristić, I.A. Figueroa, D. Pajić, Ž. Skoko, K. Zadro. Electronic structure and glass forming ability in early and late transition metal alloys. *Phil. Mag.* **98** (2018) 693–709.

[23] D. Ye, L. Jiahao, L. Baixin. Prediction of glass-forming ability and characterization of atomic structure of the Co-Ni-Zr metallic glasses by a proposed long range empirical potential. *J. Appl. Phys.* **111** (2012) 033521 (8pp).

[24] W.S. Lai, B.X. Liu. Glass-forming ability of the Ni–Zr and Ni–Ti systems determined by interatomic potentials. *J. Mater. Res.* **16** (2001) 446–450.

[25] T. Abe, H. Onodera, M. Shimono, M. Ode. Thermodynamic Modeling of the Undercooled Liquid in the Ni–Zr System. *Mater. Trans.* **46** (2005) 2838–2843; T. Abe, M. Shimono, M. Ode, H. Onodera. Estimation of the glass forming ability of the Ni–Zr and the Cu–Zr alloys. *J. Alloys Comp.* **434/435** (2007) 152–155.

[26] T. Tokunaga, S. Matsumoto, H. Ohtani, M. Hasebe. Thermodynamic Analysis of the Phase Equilibria in the Nb-Ni-Zr system. *Mater. Trans.* **48** (2007) 2263–2271.

[27] R. Bormann, F. Gärtner, K. Zöltzer. Application of the CALPHAD method for the prediction of amorphous phase formation. *J. Less-Common Met.* **145** (1988) 19–22.

[28] B.A. Klumov, R.E. Ryltsev, N.M. Chtchelkatchev. Structure and glass-forming ability of simulated Ni-Zr alloys. (2018) `arXiv:1805.05113` (11pp).

[29] T.Q. Wen, L. Tang, Y. Sun, K.M. Ho, C.Z. Wang, N. Wang. Crystal genes in a marginal glass-forming system of $Ni_{50}Zr_{50}$. *Phys. Chem. Chem. Phys.* **19** (2017) 30429–30438.

[30] I. Kaban, P. Jóvári, V. Kokotin, O. Shuleshova, B. Beuneu, K. Saksl, N. Mattern, J. Eckert, A.L. Greer. Local atomic arrangements and their topology in Ni–Zr and Cu–Zr glassy and crystalline alloys. *Acta Mater.* **61** (2013) 2509–2520.

[31] W.H. Wang, J.J. Lewandowski, A.L. Greer. Understanding the glass-forming ability of $Cu_{50}Zr_{50}$ alloys in terms of a metastable eutectic. *J. Mater. Res.* **20** (2005) 2307–2313.

5.2 Further reading

For the benefit of the reader interested in some additional information on the topics discussed in the corresponding article, we have compiled a number of selected references, which, although not being explicitely cited in the main manuscript or the supplementary material, seem to be of general interest for putting our results in the right perspective.

Selected literature on the Ni–Zr system (crystalline and amorphous phases) and NiZr twinning

[32] P. Nash, C. S. Jayanth. The Ni–Zr (Nickel-Zirconium) System. *Bull. Alloy Phase Diagr.* **5** (1984) 144–148.

[33] J.C. Barbour, R. de Reus, A.W. van der Gon, F.W. Saris. The role of diffusion in amorphous-phase formation and crystallization of amorphous NiZr. *J. Mater. Res.* **2** (1987) 168–172.

[34] Y.C. Feng, K.H. Kuo, Z.K. Hei, Y.K. Wu. A transmission electron microscopy study of the microtwins formed during the crystallization of an amorphous $NiZr_2$ alloy. *Phil. Mag. A* **56** (1987) 757–766.

[35] R. Morel, Y. Huai, R.W. Cochrane. Resistivity and Hall effect in sputtered NiZr metallic glasses. *J. Appl. Phys.* **64** (1988) 5462–5464.

[36] R. Morel, L. Abadli, R.W. Cochrane. Hall effect of sputtered NiZr and CoZr metallic glasses near the critical magnetic concentration. *J. Appl. Phys.* **67** (1990) 5790–572.

[37] I. Groma, J. Lendvai, A. Cziraki, I. Gerocs, B. Fogarassy, I. Kovacs. Crystallization of amorphous $Ni_{50-x}Cu_xZr_{50}$ alloys. *Scripta Met. Mat.* **26** (1992) 255–260.

[38] Q.-K. Li, M. Li. Surface structure and properties of NiZr model metallic glasses: A molecular dynamics simulation. *J. Non-Cryst. Solids* **354** (2008) 2060–2065.

[39] D. Holland-Moritz, S. Stüber, H. Hartmann, T. Unruh, T. Hansen, A. Meyer. Structure and dynamics of liquid $Ni_{36}Zr_{64}$ studied by neutron scattering. *Phys. Rev. B* **79** (2009) 064204.

[40] M. Ghidelli, A. Volland, J.-J. Blandin, T. Pardoen, J.-P. Raskin, F. Mompiou, P. Djemia, S, Gravier. Exploring the mechanical size effects in $Zr_{65}Ni_{35}$ thin film metallic glasses. *J. Alloys Comp.* **615** (2014) S90-S92.

[41] M. Ghidelli, S. Gravier, J.-J. Blandin, T. Pardoen, J.-P. Raskin, F. Mompiou. Compositional-induced structural change in Zr_xNi_{100-x} thin film metallic glasses. *J. Alloys Comp.* **615** (2014) S348–S351.

[42] M. Ghidelli, S. Gravier, J.-J. Blandin, P. Djemia, F. Mompiou, G. Abadias, J.-P. Raskin, T. Pardoen. Extrinsic mechanical size effects in thin ZrNi metallic glass films. *Acta Mater.* **90** (2015) 232–241.

Selected literature on the Ti-Ni-Zr quasicrystal

[43] A. Sadoc, J.Y. Kim, K.F. Kelton. Local atomic order in icosahedral Ti-Zr-Ni and hydrogenated Ti-Zr-Ni quasicrystals. *Phil. Mag. A* **79** (1999) 2763–2772.

[44] A. Sadoc, J.Y. Kim, K.F. Kelton. Local atomic structure of icosahedral quasicrystals and 1/1 approximant in the TiNiZr alloy system. *Mater. Sci. Eng.* **294–296** (2000) 348–350.

[45] A. Sadoc, E.H. Majzoub, V.T. Huett, K.F. Kelton. Evolution of the local structure with hydrogenation in Ti-Zr-Ni quasicrystals and approximants. *J. Phys.: Condens. Matter* **14** (2002) 6413–6426.

[46] A. Sadoc, E.H. Majzoub, V.T. Huett, K.F. Kelton. Local structure in hydrogenated TiZrNi quasicrystals and approximants. *J. Phys.: Condens. Matter* **356–357** (2003) 96–99.

[47] A. Sadoc, V.T. Huet, K.F. Kelton. Icosahedral ordering in Ti-Hf-Ni alloys? *J. Non-Crys. Solids* **353** (2007) 3689–3692.

Selected literature on (tenfold) twinning

[48] H. Wondratschek, W. Jeitschko. Twin domains and antiphase domains. *Acta Cryst. A* **32** (1976) 664.

[49] L. Pauling. Apparent icosahedral symmetry is due to directed multiple twinning of cubic crystals. *Nature* **317** (1985) 512–514.

[50] K. K. Fung, X. D. Zou, C. Y. Yang. Transmission electron microscopy study of $Al_{13}Fe_4$ tenfold twins in rapidly cooled Al–Fe alloys. *Phil. Mag. Lett.* **55** (1987) 27–32.

[51] X. D. Zou, K. K. Fung, K. H. Kuo. Orientation relationship of decagonal quasicrystal and tenfold twins in rapidly cooled Al–Fe alloy. *Phys. Rev. B* **35** (1987) 4526–4528.

[52] H. Zhang, D.H. Wang, K.H. Kuo. Quasicrystals, crystalline phases, and multiple twins in rapidly solidified Al-Cr alloys. *Phys. Rev B* **37** (1988) 6220–6225.

[53] H. Zhang, D.H. Wang, K.H. Kuo. Icosahedral and decagonal quasicrystals, crystalline phases, and multiple twins in rapidly solidified $Al_{13}Cr_4Si_4$. *J. Mat. Science* **24** (1989) 2981–2986.

[54] N. Wang, H. Chen, K.H. Kuo. 45° twins with apparent eightfold symmetry in $Cr_5Ni_3Si_2$ alloy. *Phil. Mag. B* **60** (1989) 347–363.

[55] M. Ellner, U. Burkhardt. Zur Bildung von Drehmehrlingen mit pentagonaler Pseudosymmetrie beim Erstarrungsvorgang des Fe_4Al_{13}. *J. Alloys Comp.* **198** (1993) 91–100.

[56] M. Ellner, U. Burkhardt. Multiple twinning of Fe_4Al_{13} showing pentagonal pseudosymmetry. *Mat. Sci. Forum* **150–151** (1994) 97–108.

[57] M. Ellner. Polymorphic phase transformation of Fe_4Al_{13} causing multiple twinning with decagonal pseudosymmetry. *Acta Cryst.* B**51** (1995) 31–36.

[58] J. Wang, B. Zhang, Y. T. Zhou, X. L. Ma. Multiple twins of a decagonal approximant embedded in S-Al_2CuMg phase resulting in pitting initiation of a 2014Al alloy. *Acta Mater.* **82**, 22–31.

[59] J. Wang, B. Zhang, Z. B. He, B. Wu, X. L. Ma. Atomic-scale mapping of twins and relevant defective structures in $Al_{20}Cu_2Mn_3$ decagonal approximant. *Phil. Mag.* **96**, 2457–2467.

Selected literature on multiple twinning

[60] H. Hofmeister. Forty Years Study of Fivefold Twinned Structures in Small Particles and Thin Films. *Cryst. Res. Technol.* **33** (1998) 3–25.

[61] H. Hofmeister. Shape variations and anisotropic growth of multiply twinned nanoparticles. *Z. Kristallogr.* **224** (2009) 528–538.

Selected literature on liquid metals and metallic glasses

[62] J. D. Bernal. Geometry of the structure of monatomic liquids. *Nature* **185** (1960) 68–70.

[63] J. D. Bernal. The Structure of Liquids. *Proc. Royal Soc. Lond. A* **280** (1964) 299–322.

[64] T. Schenk, D. Holland-Moritz, V. Simonet, R. Bellisent, D. M. Herlach. Icosahedral Short-Range Order in Deeply Undercooled Metallic Melts. *Phys. Rev. Lett.* **89** (2002) 075507.

[65] Q. Wang, L.M. Wang, M.Z. Ma, S. Binder, T. Volkmann, D. M. Herlach. Diffusion-controlled crystal growth in deeply undercooled melt on approaching the glass transition. *Phys. Rev B* **83** (2011) 014202.

[66] C. Tang, P. Harrowell. Anomalously slow crystal growth of the glass-forming alloy CuZr. *Nature Materials* **12** (2013) 507–511.

Selected literature on nucleation

[67] A. Mariaux, M. Rappaz. Influence of anisotropy on heterogeneous nucleation. *Acta. Mater.* **59** (2011) 927–933.

[68] M. Rappaz, J. Friedli, A. Mariaux, M. Salgado-Ordorica. The influence of solid-liquid interfacial energy anisotropy on equilibrium shapes, nucleation, triple lines and growth morphologies. *Sripta Mater.* **62** (2010) 904–909.

Selected literature on twinned dendrites

[69] K. Chattopadhyay, S. Lele, P. Ramachandrarao. On the occurence of twinned dendrites in rapidly solidified aluminium alloys. *J. Cryst. Growth* 49 (1980) 322–324.

[70] A.M. Mullis, K.I. Dragnevski, R.F. Cochrane. The solidification of undercooled melts via twinned dendritic growth. *Mater. Sci. Eng. A* 375–377 (2004) 547–551.

[71] M.A. Salgado-Ordorica, J. Valloton, M. Rappaz. Study of twinned dendrite growth stability. *Scripta Mater.* **61** (2009) 367–370.

Selected literature on miscellaneous topics

[72] E. Boehm-Courjault *et al.* EBSD: a powerful microstructure analysis technique in the field of solidification. *J. Microsc.* **233** (2009) 160–169.

[73] K.H. Kuo. Mackay, anti-Mackay, double-Mackay, pseudo-Mackay, and related icosahedral shell clusters. *Struct. Chem.* **13** (2002) 221–230.

[74] T. Weber *et al.* Large, larger, largest - a family of cluster-based tantalum copper aluminides with giant unit cells. I. Structure solution and refinement. *Acta Cryst. B* **65** (2009) 308–317.

[75] S. Andersson, L. Stenberg. Cyclic intergrowth and crystal structures. *Z. Kristallogr.* **158** (1982) 133–139.

[76] D. Hohnke, E. Parthé. *AB* Compounds with Sc, Y and Rare Earth Metals. II. FeB and CrB Type Structures of Monosilicides and Germanides. *Acta Cryst.* **20** (1966) 572–582.

[77] W. Wisniewski, M. Seyring, C. Patzig, T. Höche, A. Keshavarzi, C. Rüssel. Bulk Crystallization in a $SiO_2/Al_2O_3/Y_2O_3/AlF_3/B_2O_3/Na_2O$ Glass: Fivefold Pseudo Symmetry due to Monoclinic Growth in a Glassy Matrix Containing Growth Barriers. *Sci. Rep.* **6** (2016) 19645.

[78] W. Steurer, S. Deloudi. Decagonal quasicrystals – What has been achieved? *C. R. Physique* **15** (2014) 40–47.
